# Understanding the role of self-determination in shaping university experiences for autistic and typically developing students in the United Kingdom

**DOI:** 10.1177/1362361320984897

**Published:** 2021-02-03

**Authors:** Jiedi Lei, Ailsa Russell

**Affiliations:** 1 Centre for Applied Autism Research, Department of Psychology, University of Bath, Bath, UK; 2 King’s College London, Department of Psychology, Institute of Psychiatry Psychology & Neuroscience, De Crespigny Park, London, UK

**Keywords:** autism spectrum disorder, autonomy, college, competence, relatedness, self-determination, university

## Abstract

With more autistic students enrolling in higher education, little is known about how autistic students can actively and effectively shape their own university experience through self-determination. This study explores how both autistic (*n* = 18) and typically developing students and recent graduates (*n* = 18) perceive their self-determination during their transition *into, through* and *out of* university in the United Kingdom. Students reported many shared and unique aspects of autonomy, competence and relatedness underlying self-determination. Many autistic students also discussed autism-related strengths facilitating academic pursuit at university, though found coping with transitional changes more difficult than typically developing students. Using strength-based approaches to help autistic students to actively adapt to routine changes might facilitate their self-determination during transition to university.

**Lay abstract:**

Prior research suggests that autistic students in higher education might struggle with developing autonomy, competence and establish relatedness due to their executive functioning and social communication difficulties. We interviewed 18 autistic and 18 typically developing students to explore how students perceived themselves to be in control of their university experience. Both groups provided anecdotal examples that supported similar perceptions of self-determination in shaping the academic, daily living and socialisation aspects of university life. Autistic students reflected on their cognitive strengths such as attention to detail, persistence and ability to tailor their academic studies towards their interest. Varying degrees of sociability were noted, with some autistic students preferring to focus their self-determination efforts on academic success, while others treasured the novel social experiences including peer support and friendship at university. Compared to greater flexibility endorsed by typically developing students, autistic students perceived establishing a routine at university to be a necessity and were self-determined in maintaining stability amid a sea of change. Recognising strengths and self-determination efforts in autistic students can help stakeholders support their personal development towards independent living and self-sufficiency in adulthood and to successfully transition *into, through* and *out* of university.

For young people, university can be a springboard to a multitude of social and academic opportunities depending on one’s ability to effectively shape their experience through self-determination (Field et al., 2003). Although self-determination has been identified as an important construct related to university transition for autistic students (Field & Hoffman, 1999; Wehmeyer et al., 2010), little is known about how autistic students’ perception of their own self-determination through a first-person perspective may compare to their neurotypical peers. To the best of our knowledge, this is the first qualitative study to explore how autistic and neurotypical students perceive their own self-determination transitioning *into, through* and *out of* university. Focus is paid to students’ experience across academic, daily living and socialisation domains of their university lives. Similarities and differences in autistic and neurotypical students’ perception of their ability to shape their university experience are highlighted.

## Self-determination and higher education

Self-determination is conceptualised as the inherent human tendency towards psychological growth, independence and improved wellbeing, based on meeting the basic needs of autonomy, competence and relatedness (Deci & Ryan, 1985; Ryan & Deci, 2000; Wehmeyer, 2005). Autonomy refers to one’s ability to self-regulate and initiate actions; competence refers to having the knowledge, skills and understanding to achieve desirable outcomes congruent with one’s goals; and relatedness refers to the development of a secure and satisfying social network (Deci et al., 1991). Cognitive evaluation theory (Deci et al., 1991) further stated that compared to intrinsically motivated behaviours, extrinsically motivated behaviours can only become self-determined if the external goals are congruent with one’s internal values and sense of self, and such integration can foster a sense of belonging and connectedness towards someone they value (i.e. through relatedness) (Gagné & Deci, 2005).

To date, quantitative studies have documented that typically developing (TD) students’ perceived sense of autonomy and competence are predictors of academic success and enjoyment at university (Black & Deci, 2000; Goldman et al., 2017; Liu et al., 2014). Self-determination of TD students also underlies the pursuit of both intrinsically or extrinsically motivated actions (Niemiec & Ryan, 2009). However, the use of qualitative methods to explore TD students’ voices and perceptions of self-determination at university are lacking. In contrast, qualitative methods are more widely used to examine the relationship between self-determination and university success among students with learning disabilities and specific learning difficulties (Field et al., 2003; Getzel & Thoma, 2008; Ju et al., 2017; Petcu et al., 2017; Sarver, 2000). Using qualitative methods, many attributes, such as problem-solving skills, persistence, being aware of one’s strengths and weaknesses, setting appropriate short- and long-term realistic goals and self-management, were identified as key self-determination skills that an effective self-advocate would have to access support and succeed in postsecondary education (Getzel & Thoma, 2008; Sarver, 2000). Educators may therefore facilitate self-determination among both student groups by helping them identify and internalise extrinsically motivated goals of academic performance through fostering a collaborative and supportive learning environment at university (Black & Deci, 2000; Goldman et al., 2017; Hong et al., 2011) and ensure student voices are being heard (Niemiec & Ryan, 2009). For students with disabilities, developing support systems through meaningful relationships with peers and professors also contributed towards college retention, highlighting that relatedness in addition to autonomy and competence facilitates self-determination in higher education (Getzel & Thoma, 2008).

## Autism and self-determination

Autism spectrum disorder (ASD) is a neurodevelopmental condition characterised by social and communication difficulties and restricted and repetitive behaviours and interests (American Psychiatric Association, 2013). A rise in UK higher education attendance by autistic^1^ individuals reflects recent movements in neurodiversity and widening participation (MacLeod & Green, 2009). According to data reported by Office for Students (2019), the number and relative percentage for students with social or communication impairment (including ASD) have risen from 2465 (0.2%) students in 2010-2011 to 10,890 (0.7%) in 2018–2019. Given that many autistic students experience co-occurring conditions, a proportion of them may be captured by data including students with multiple impairments (including social/communication impairment, sensory, medical, physical and mental health conditions) which also showed an increase from 30,955 (2%) students in 2010-2011 to 44,490 (2.8%) in 2018–2019.

In a recent narrative synthesis of studies that have examined neurodiversity in higher education (Clouder et al., 2020), the authors identified the need for universities to encourage students to disclose their diagnosis before they reach a crisis point and can no longer cope with the demands of university life (Van Hees et al., 2015). Diagnosis disclosure enables students to access practical and social/emotional support available on campus, as well as for reasonable adjustments to be made (Clouder et al., 2020). However, the quality of support and adjustments may differ depending on the knowledge and training of university staff members (Clouder et al., 2020). The authors also highlighted that despite support systems being available in higher education, students need to be more active and serve as their own advocates when approaching the university to seek out support tailored to their needs, thus suggesting that the role of self-determination and agency may play an important role in their ability to shape their own university experience (Clouder et al., 2020).

Self-determination is therefore important to consider for autistic students (Wehmeyer et al., 2010). Many autistic students might have executive functioning difficulties (e.g. poor cognitive flexibility and working memory to engage with multiple goals concurrently, time management and organisation difficulties; Dijkhuis et al., 2020) which make the actualisation of their self-determined goals harder to accomplish. Parents of autistic young people have noted that performance and capacity for skills related to self-determination (e.g. problem-solving, self-management, decision-making) for this group of young people were low (Carter et al., 2013). Compared to students with specific learning difficulties and learning disabilities, autistic middle and high school students showed poorer autonomy, self-regulation, psychological empowerment and self-realisation, suggesting that social communication difficulties unique to autism might place them at a particular disadvantage relative to their peers with other disabilities in establishing a sense of relatedness (Chou, Wehmeyer, Palmer, & Lee, 2017).

Wehmeyer et al. (2010) emphasised that educators should encourage autistic students to self-advocate for their strengths and needs, to improve their self-regulation and flexibility, set realistic goals that can be achieved and to exercise their decision-making skills whenever possible, as all are important for independent living in adulthood (Field & Hoffman, 1999). Educators may consider adopting a strength-based framework to help autistic students increase their sense of autonomy and perceived competence underlying self-determination. For example, helping autistic students recognise their own strengths such as in visual perception and attention to detail, good systemising skills and having strong interests and in-depth knowledge of certain fields can enable them to become more confident in their own competence (de Schipper et al., 2016; Lee et al., 2019; Urbanowicz et al., 2019), especially when pursuing academic success (Bakker et al., 2019). Helping students recognise how they can best harness and utilise their own strengths to overcome problems in their university lives (beyond the academic domain) can further enable them to experience a greater sense of autonomy and empowerment and help students live more authentically as self-fulfilling agents with better quality of life (Lee et al., 2019; Urbanowicz et al., 2019).

To date, there is quantitative evidence to support a positive association between better quality of life and autonomy, psychological empowerment, self-realisation and having the capacity to become self-determined in autistic young adults (White et al., 2018). However, it remains unknown from a firsthand qualitative perspective if and how autistic students perceive themselves to be effective in shaping their own university experience through self-determination compared to TD peers who have similar academic background and interests.

## Current study

This is the first qualitative study to collectively investigate autistic and TD students’ reflections on the extent to which they shaped their university experiences through self-determination. Breaking down university experience into three stages, namely, transitioning *into, through and out of* university, we examined whether students demonstrated evidence for autonomy, competence and relatedness in their experiences. Given that prior literature suggested that autistic students find self-determination more challenging than others due to their social communication difficulties and unique cognitive styles, we selected a group of TD students matched to autistic students based on age, sex, pre-academic performance level, and degree subjects studied at university to investigate both shared and unique experiences related to self-determination between autistic and TD students.

## Methods

### Student sample selection and characteristics

A total of 36 students (18 TD and 18 autistic students) took part in the study. Students’ demographic and diagnostic information are displayed in Table 1. Students were recruited through flyers and student groups on social media channels advertised to university students throughout the United Kingdom. For the autism group, students were included based on their self-report of having received a formal diagnosis of autism from a clinical professional and have disclosed their clinical diagnosis to their university disability service and are eligible to access autism-specific support on campus. Some students may have received clinical diagnosis during childhood prior to the publication of *Diagnostic and Statistical Manual of Mental Disorders (5th ed., DSM-5*) in 2013 which introduced the umbrella term ASD, and we report the diagnostic label provided to us by students. Sixteen out of 18 autistic students met screening cut-off on the autism quotient (AQ), 12 of whom also met clinical cut-off. Many autistic students experienced at least one co-occurring mental or chronic physical health conditions, or specific learning disability, the most prevalent being anxiety, depression and specific learning disability.

**Table 1.** Student demographic information.

	ASD (*n* = 18)	TD (*n* = 18)
	*M* (*SD*)	
Age (years)	20.94 (3.24)	19.83 (1.38)
Gender	(*n*, %)	
Male	9 (50)	9 (50)
Female	9 (50)	9 (50)
Ethnicity		
White/Caucasian	16 (88.89)	13
Asian	0	3
Two or more races	2 (11.11)	1
Prefer not to say	0	1
Autism diagnosis		
Autism/ASD	5 (27.78)	–
Asperger’s	12 (66.67)	–
PDD-NOS	1 (5.56)	–
Other diagnoses		
Anxiety	6 (33.33)	–
Depression	5 (27.78)	–
ADHD	3 (16.67)	–
Specific learning disability	6 (33.33)	–
Anorexia Nervosa	1 (5.56)	–
Other medical conditions	2 (11.11)	–
Pre-university qualifications	*M* (*SD*)	
Number of A-levels^a^ (or equivalent) completed	4 (1.37)	4.39 (0.92)
Average grade^b^	4.34 (1.24)	5.07 (0.91)
Subject/degree at university	(*n*, %)	
Social sciences	7 (38.89)	8 (44.44)
Arts and humanities	4 (22.22)	3 (16.67)
STEM	7 (38.89)	7 (38.89)
Living status		
On campus	6 (33.33)	9 (50)
Off campus with family	6 (22.22)	0
Off campus with peers	6 (44.44)	9 (50)
Current year of study		
First	7 (38.89)	5 (27.78)
Second	7 (38.89)	7 (38.89)
Fourth/final	2 (11.11)	2 (11.11)
Have graduated already	2 (11.11)	4 (22.22)
Autism quotient	*M* (*SD*)	
Total^c^	34.22 (9.57)	11.83 (5.72)
Interview format	(*n*, %)	
Face to face	8 (44.44)	5 (27.78)
Phone	8 (44.44)	12 (66.67)
Skype	2 (11.11)	1 (5.56)

ASD: autism spectrum disorder; TD: typically developing; PDD-NOS: pervasive developmental disorder – not otherwise specified; ADHD: attention deficit hyperactivity disorder; STEM: Science, Technology, Engineering, Mathematics.

a In the UK, students typically complete 3–5 A-levels in chosen subjects final year of secondary school, before entering university.

b Each A-level (or equivalent) is scored on a scale of 0 (fail) to 6 (A* = highest grade).

c The clinical cut-off score for the autism quotient (AQ) is ⩾32 and screening cut-off is ⩾26.

For the TD group, students were only included if they reported no current or past diagnoses for mental health, chronic physical illness or any other forms of specific learning difficulty or developmental condition. Given that this study is the first to explore autistic and TD students’ shared experiences in self-determination at university, we chose to include only TD students with no current or past diagnoses to reflect upon their university experiences that was not impacted by having any mental or physical health condition(s). Therefore, the challenges that TD students reported may reflect general challenges associated with transitioning *into, through and out of* university for most students, rather than unique challenges due to any personal health conditions. This allowed us to gain insight into how students may act in a self-determined way when addressing typical daily challenges at university. TD students were excluded if they scored above the screening cut-off on the AQ. Neither the TD students met screening cut-off on the AQ nor they had any current or past mental health, chronic physical health or specific learning conditions.

All students must have either attended or currently attending undergraduate studies in the United Kingdom and spoke fluent English. Autistic and TD students were matched on gender, age (*t*(34) = 1.34, *p* = 0.19) and pre-university academic performance, both in terms of number of A-levels (or equivalent) completed (*t*(34) = −1, *p* = 0.32), mean grade received for all A-levels competed (*t*(34) = −2.02, *p* = 0.05). Students in the TD group came from ten different institutions and autistic students came from eleven different institutions in the United Kingdom. In terms of living status, one-third of autistic students lived at home with family, compared to TD students who either lived on campus or off campus with peers. We did not record specific information on socioeconomic status in this study.

### Ethical consideration

The study was approved by the university’s Psychology Department Ethics Committee and performed in accordance with the ethical standards of the institution and the 1964 Helsinki declaration and its later amendments. All students provided individual written informed consent prior to participating in the study.

### Materials

See Appendix 1 for information on demographic questionnaire and AQ (Baron-Cohen et al., 2001).

#### Interview topic guide

The first author developed the interview topic guide (see Appendix 2) with the aim of gaining insight into students’ experiences at university through their own narrative account. Taking a critical realist approach, we were interested in whether students may describe their personal experience in a way that naturally reflected a sense of autonomy, competence and relatedness without explicitly being asked to provide evidence for each of the three domains of self-determination. Therefore, we asked students to reflect upon their experience of transitioning *into, through* and *out of* university (or for those yet to graduate, conjecture what life after university might be like). We designed the questions to be more open to allow students to recall aspects of university life perceived to be most important to themselves. In order for the interview to capture students’ sense of agency, the interviewer asked students to think about to what extent they have shaped their academic, daily living and socialisation life at university. Students reflected on how their experiences might have compared to others and whether there were things that they had wished to be different. Finally, students reflected upon whether they have gained any skills during university that might be helpful for the future. For autistic students, one question specifically prompted them to think about the impact of autism on their university experience, to ensure that there is scope to capture how autism associated strengths and weaknesses may have shaped their university experience.

### Community involvement

One autistic graduate student helped to pilot the interview guide to see whether the questions could elicit recall of their university experience across academic, daily living and social domains and to ensure that the interview length was appropriate. The same student also provided feedback on ways to improve the clarity of the wording to minimise the chance of misinterpreting the questions during the interview. Autistic students did not participate in the research design, analysis or interpretation of findings in this study.

### Procedure

All students interested in taking part read through the study information, completed written informed consent and filled in an online questionnaire (basic demographic information and AQ) via Qualtrics. Students who met the inclusion criteria and successfully completed the online questionnaires were offered to attend the interview in person, via phone or Skype. The latter options were offered given that many students lived and attended institutions that far away from the research team and students preferred to conduct the interview remotely rather than travelling to do so in person. Interviews lasted 20–45 min, and students received £10 Amazon gift vouchers upon completing the interview.

### Analysis

We conducted thematic analysis following the Braun and Clarke (2006, 2013) method. We adopted a critical realist approach and focused on the semantic features of the interview data. We first adopted a deductive approach when examining all transcripts to assess whether there may be evidence that support the notion of self-determination as outlined by Ryan and Deci (2000) (i.e. autonomy, competence and relatedness). Referring to critical realism, the first step served to critically evaluate whether observations made in the ‘experiential’ and ‘actual’ spheres (i.e. students’ perception of their personal experience as well as the events and actions they took during their time at university) may be related to the ‘real’ (i.e. whether autonomy, competence or relatedness underlying self-determination served as potential causal mechanisms that influenced students’ ability and desire to shape their university experience).

We analysed data from both autistic and TD students as a whole rather than split into two groups, as we wanted to highlight how university students reflected upon their self-determination at university in general. The nuanced differences at the level of each subtheme raised by autistic students are highlighted to showcase their additional perspectives beyond that of TD students. The first author who conducted all of the interviews familiarised herself with the data through transcribing, reading and re-reading of the transcript, and developed initial codes which were then returned to, revised and evaluated together with the senior author who is an experienced autism researcher and clinical psychologist.

The first author identified relevant excerpts across all transcripts that provided evidence supporting self-determination and sorted them into three bins which corresponded to autonomy, competence and relatedness. The first author broke down excerpts that supported more than one of the three constructs so that the relevant sections for each construct can be analysed independently. For excerpts that supported more than one construct and could not be broken down further, the semantic content of the excerpt was interpreted within the context of each specific construct and coded accordingly. The first author took an inductive approach when analysing data within each bin and coded semantically to best characterise key features present in the data.

During finalisation of the codes, both authors first ensured that codes and themes were sufficiently distinct from each other *within* each of the three bins and remained characteristic of the three domains of autonomy, competence and relatedness underlying self-determination. We then compared the themes *across* the three different bins, to examine whether certain themes may be discussed within more than one of the three pillars underlying self-determination (i.e. relying on a combination of autonomy, competence and relatedness). This final step allowed us to distinguish between themes that were unique to each of the three domains underlying self-determination and themes that were common across multiple domains. We expected there to be some overlap in the way that students recounted their university experience to show that they fulfilled more than one of the three needs underlying self-determination (Ryan & Deci, 2000).

The first author was not blinded to students’ diagnosis during the interviews or analyses, though data were collated across the two student groups to be analysed together, and codes were not created to be autism specific. We did not use a second rater to assess inter-rater reliability in the initial steps of coding as a measure of quality in this study. Given that the first author conducted all of the interviews in an interactive manner using a topic guide rather than in a semi-structured way, her interactions with the students informed the subsequent coding and understanding of the data in a way that a second rater would be unable to replicate (Morse, 1997). Therefore, the use of a second rater would be incoherent with the current epistemological and ontological positions adopted (Braun & Clarke, 2006, 2013; Terry & Braun, 2016).

## Results

Interview format and duration information are shown in Table 1 and Appendix 1.

### Thematic analyses

**Figure 1.**
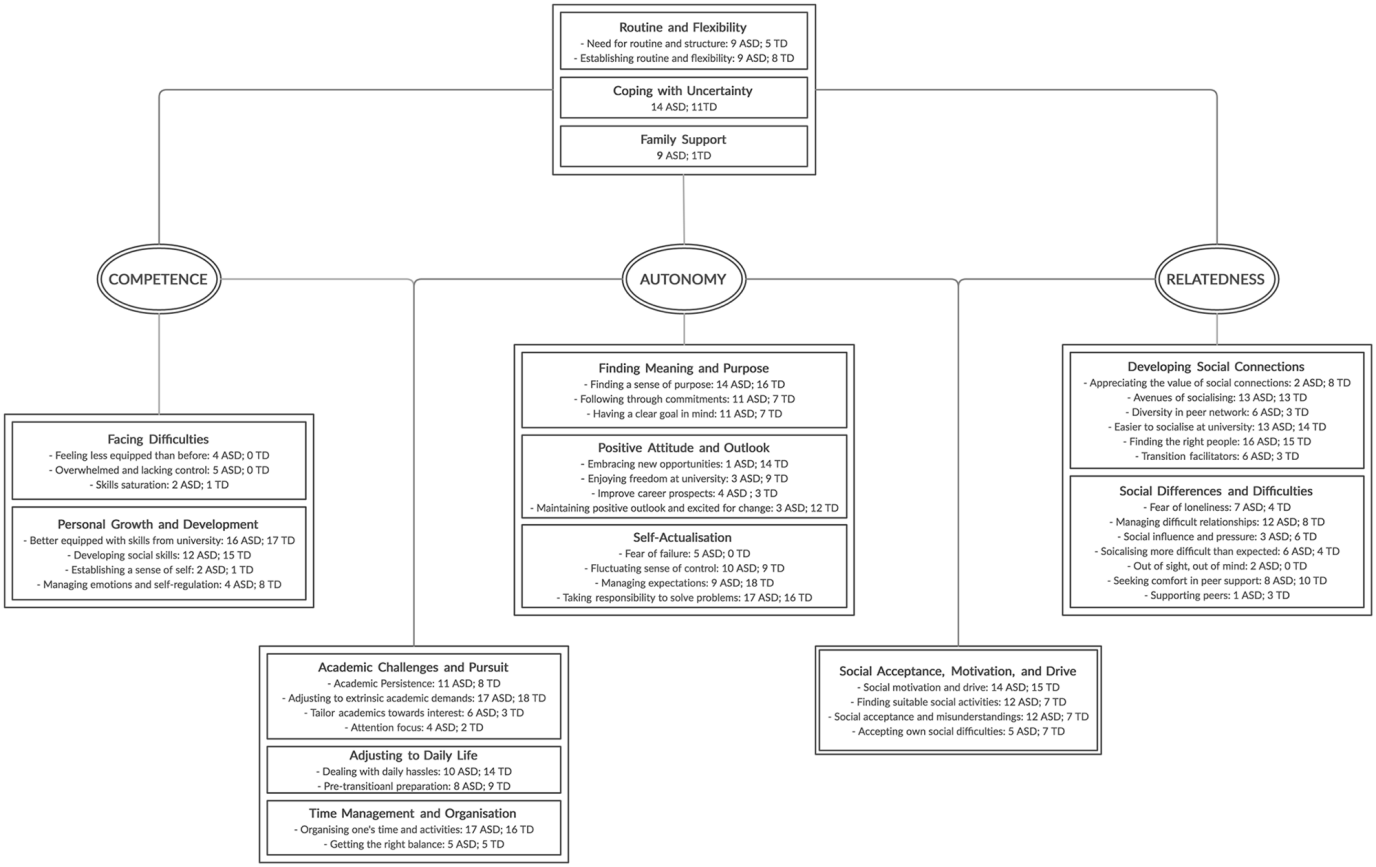
Thematic analyses output map identifying themes that are unique to or shared between autonomy, competence and relatedness. The number of autistic (*n* = 18) and TD (*n* = 18) students who endorsed each theme are provided.

Themes and subthemes identified as unique to or shared across autonomy, competence and relatedness are shown in the thematic map (Figure 1). We quantified endorsement by students (Figure 1) post hoc (i.e. upon completing the development and selection of themes during thematic analyses). We coded endorsement in a binary sense, based on whether the student referred to that theme (1) at least one time or not (0) in their transcript. Therefore, the number provided can be interpreted as a headcount for the number of students that endorsed each theme. Given that this study included both a group of TD and autistic students, the sole purpose for providing this quantitative comparison is to characterise potential differences in the extent to which each student group related to the specific themes, supplementing the results from the thematic analysis. The quantification should not be interpreted as a guide for the relevance of or used to rank the importance of the themes across the different domains of self-determination, as this is advised against by the thematic analysis approach outlined by Braun and Clarke (2006, 2013). We selected quote(s) to represent students’ general and nuanced opinions. To protect students’ anonymity, we took precautions to remove or modify any potential identifiable information from the selected quotes. We first describe similarities across both student groups when discussing ideas related to each theme and then highlight between-group differences by outlining ways in which autistic students may have had additional or different insight when describing their own experiences. Please refer to Figure 1 as a guide for the overall thematic structure when reading through the results below.

#### Autonomy

We identified three themes from students’ responses that uniquely reflected autonomy.

##### Finding meaning and purpose

Despite being determined to achieve their goals, students had uncertainties as to what that goal might be and how it might change over time, especially juggling the social and academic aspects of university life:

I think that for me now it’s less towards getting the top grade. . . I guess that goal then kind of became more, just kind of wanting to have good personal connections and just be happy. (P11, ASD)

When thinking about the future, students reflected on the lack of a clearly defined path when transitioning out of university. When talking about their degrees, TD students were less likely to draw upon the distinction between intrinsically and extrinsically motivated goals, autistic students spoke about being motivated only to pursue goals that naturally aligned with their intrinsic interests:

If I’m not interested in something, then I just can’t really be bothered, I don’t have that motivation, I don’t want to do it. . . (P06, ASD)

Similarly, for future career prospects, TD students were more flexible in the career paths that they will pursue:

I knew psychology was what I wanted to do, but at the same time I was still being flexible, like those goals could change. . . I could find a different route to those goals sort of thing. (P29, TD)

In contrast, autistic students were more fixated on specific professions that were related to their intrinsic interest and the degree they were pursuing.

##### Positive attitude and outlook

Students talked about trying out new things to broaden their experience and enjoying the freedom to schedule their own time at university when compared to school and agreed that a good degree would improve career prospects. Autistic students were less likely to engage with and described difficulties accessing extracurricular activities, which they perceived to lack usefulness. TD students described excitement and readiness for change both when transitioning *into* and *out* of university:

I actually remember not even being actually that nervous which is strange, because obviously it was such a big thing. . . I just remembering being really excited about it, excited to start, meet new people as well. (P37, TD)

While autistic students had a less positive outlook towards transitions, in general:

I suppose I’m always maintaining this hope that things are going to get better, but that’s probably just a defence mechanism. (P03, ASD)

##### Self-actualisation

Students perceived self-control as more of a ‘state than a trait’. Self-control varied depending on the area of university life and sometimes led to counter-productive decisions being made and in the absence of self-discipline. Students noted the importance of being able to manage one’s own expectations through recognising personal strengths and weaknesses and not comparing self to others to maintain mental wellbeing at university. Following advice from others can set up false expectations of what university might be like, which resulted in disappointment when compared to reality:

You have to kind of lower your expectations, lower your competitiveness, because a lot of people went from top of the school, or quite high up in their school, and now they are average at university, and preparing for that was also quite hard. (P33, TD)

Finally, students talked about a growing sense of independence, accountability and responsibility when it comes to problem-solving at university:

Everything depends on you, and actually you have to do it yourself, because if you don’t do it you just don’t do it, no one will punish you, but no one will help you at the same time. (P25, TD)

Compared to TD peers, autistic students expressed more conflicting opinions regarding problem-solving, as some wanted to do things independently and others felt more comfortable seeking support from others who were willing to help. Many spoke of an intense fear of failing one’s academics at university, which can both be highly motivating to secure academic success (sometimes at the cost of socialising), but can also immobilise one’s desire to try harder as it can be rather disappointing if one does not succeed.

#### Competence

We identified two themes from students’ responses that uniquely related to competence.

##### Facing difficulties

Students commented on low motivation to complete university when they perceived a lack of personal development. Autistic students felt less equipped both socially and academically to cope with the demands at university compared to their TD peers, and this lack of direction and competency contributed towards feeling overwhelmed and inability to stay in control:

I felt completely lost. . . School to university was like falling off a cliff. Going. . . well when I went to work it felt. . . I don’t know. . . going for a walk on some hills, there were ups and downs but overall it was a lot more even than a cliff face. (P18, ASD)

##### Personal growth and development

Students spoke about developing some new hard (academic and technical) and soft (inter/intrapersonal) transferrable skills at university, which they foresaw would help them in the future:

I think at uni they’ve like equipped us with a sort of confidence to sort of then go out and feel like we can do a job and do well at it. (P23, TD)

Students described being able to adopt a new perspective when faced with difficult situations, indicating growth and development. Akin to a journey of self-discovery, students learnt to present themselves in an authentic way over time. Students described feeling more comfortable to meet and learn from new people. Autistic students talked about an improvement in managing social naïveté in university with a shift in attitude to actively work on one’s sociability:

The having to practice talking to people and cooperating with people because there’s so much group work, and be diplomatic and stuff, I think that will be useful in the future because you have to get on with people. (P05, ASD)

Contrastingly, while TD students spoke about learning new strategies to cope with anxiety and stress, autistic students described feeling less able to regulate their own emotions:

I think the balance between doing things and learning new things, and having these opportunities, and kind of the anxiety that comes with it, and knowing that you can enjoy it, is a really hard balance that I struggle with. (P11, ASD)

#### Relatedness

We identified two themes from students’ responses that uniquely related to relatedness.

##### Developing social connections

Students talked about their appreciation of having made important social connections at university through both formal academic settings and informally through shared interests and societies. Such friendship provided a source of companionship and support and is an important part of university life:

Now that I’ve got this whole community of different people, it’s so wonderful to have such a supportive group of friends, and have peers who kind of band together to help each other with their projects. (P15, ASD)

Students expressed a desire to diversify their social network so that not all friends are from the same group, which can be difficult to manage at times and appear ‘cliquey’. Students found it easier to socialise at the start of university, as everyone tried to make friends by being open and friendly, though there was a distinction between peers they simply got along with, versus peers that they ‘clicked’ with and became good friends, and thus finding the right group of people was deemed to be important. Students talked about significant people in their lives, such as a helpful lecturer, or older students who provided reassurance to help ease into the transition, and using social media channels to familiarise themselves with housemates prior to transition:

I’m still closer with the people that I knew from the start, so that just kind of built the very nice initial bond then. (P24, TD)

In addition, autistic students also remarked that having pre-arrival preparation events organised by the university provided valuable in-person meeting experiences and that university allowed them to socialise with peers outside of work-related settings.

##### Social differences and difficulties

Students found university can sometimes be quite a lonely place as socialising is not always easy, though there is a difference between being alone, feeling lonely and isolated:

I’ve never felt like I’m completely alone at uni, but sometimes if you are not getting on with your friends so well. . . or like you see them doing more things at uni, and you feel like oh that’s where I should be, or you feel like you know, I’m not in that group or something. So I’d say that sometimes you feel isolated. (P31, TD)

I am probably an awful lot more alone, and sometimes that can feel lonely. Being alone and being lonely are quite different things. (P03, ASD)

Maintaining social interactions overtime can be challenging, especially when relationships begin to breakdown due to individual differences, and the challenges of socialising and being understood by same aged peers. Students talked about how peer influence can motivate them to stay on track but can also lead to a sense of obligation when it comes to socialising, which can be exhausting:

I guess some aspects were ok, tolerable, others were slightly more tedious, like the going out, you had to intermingle and socialise, that was fairly laborious. (P16, ASD)

Although both student groups appreciated having a supportive network away from home, providing emotional support to others can be draining at times and negatively influence one’s own mental health. Autistic students also commented on the lack of motivation to initiate social interaction to actively maintain pre-university friendships when they were no longer within physical proximity at university.

#### Autonomy and competence

We identified three themes from students’ responses that related to both autonomy and competence.

##### Academic challenges and pursuit

Students found that having a good academic foundation both in terms of school preparation and learning style to prepare for university to be crucial. Students talked about enjoying the academic freedom at university to tailor subjects towards their own areas of interest. Students discussed the difficulties of adjusting to the new teaching and independent learning environment at university and that the lack of clear guidance and structure in terms of how to determine appropriate quantity and quality of workload affected their mental wellbeing and academic motivation. Both student groups emphasised the importance of persistence being the key to academic success and not being let down by failures:

It’s more about trying rather than maybe succeeding. (P42, TD)

Autistic students also reflected upon how strengths associated with autism such as tenacity and having a more detail orientated, systematic and analytical cognitive style can be used to their academic advantage. Autistic students found tailoring their academic assessments towards their special interests can lead to peaks and troughs in motivation, concentration, attention focus and work quality:

I think it’s a mixture of both positive and negative. From a positive aspect, it’s handy that I have a special interest in psychology, so that means I can definitely learn a lot quicker than most people, and I can do really well with it, which is really handy. The downside is when my special interest changes at times, and I end up thinking more on other subjects than I probably should. (P08, ASD)

##### Adjusting to daily Life

Students talked about the need to have self-discipline and organisation skills to ensure proper self-care. Students often gave daily living tasks less priority compared to social and academic tasks and left incomplete, which negatively affected students’ mental wellbeing. Students recognised the lack of independent living skills prior to university when living at home and reported to have actively tried to develop skills such as cooking, driving and budgeting in preparation for university. Students highlighted previous experiences of living away from home, whether for recreational (e.g. travelling), health (e.g. hospital stays) or academic purposes (e.g. academic summer camp) to be particularly helpful in getting used to being away from home, and not feeling homesick when transitioning to university.

##### Time management and organisation

In terms of time management, students recognised poor organisation and talked about struggling during unstructured time at university and how a lack of self-discipline can lead to continued procrastination when working towards deadlines. Students discussed strategies to help them stay on schedule, including seeking support from others, making lists and setting reminders. Students spoke about trying to strike a good work/life balance, though TD students talked about being more mindful of the bigger picture and accepting that falling behind is bound to be part of life but having the confidence that one will be able to catch up in time:

I’ve got a part time job which you need to lend time to as well, so I think it’s for me the adjustment of feeling alright with sometimes being behind on work, and that’s not the end of the world, because you can catch up, so that sort of adult life of balancing everything. (P23, TD)

In contrast, autistic students spoke about academia taking priority and willingness to sacrifice social opportunities to secure academic success:

I find that sometimes social life distracts form. . . I feel like university should be more about academia and doing well and achieving for yourself, but other people seem to value the social side more, and I just don’t really know. It’s a bit uncertain for me how to get the balance. (P03, ASD).

#### Autonomy and relatedness

We identified one theme from students’ responses that related to both autonomy and relatedness.

##### Social acceptance, motivation and drive

Students spoke about using social motivation to push oneself to socialise at university and actively trying to make friends:

. . . And I think when I realised that and I know that I get a lot of pleasure and happiness from social connections, then my kind of driving force behind creating those connections, it wasn’t kind of because I felt like I should be doing it, or because you know, for some kind of strange reason like I’m going to be missing out, it was more like this is how I know I’m going to be happy, and I guess I realised throughout, so that sort of self-determination became less academic and more social, I’ve never had social motivation before to have friends, but again I just was never really fussed, until university, I realised how good it can be. (P11, ASD)

Students talked about having social selectivity in order to manage their social time more effectively:

If I didn’t feel like I can get on with someone, or I couldn’t see that we would be friends, then I would just go and focus on the people who I knew I had a stronger bond with, so I guess in that way that was self-determining. (P32, TD)

Splitting the university social scene based on alcohol consumption, students discussed the importance of finding the right social activities, especially for non-drinkers, and feeling comfortable with one’s own decision rather than being peer-pressured into undesirable activities. Differences arose when students talked about accepting one’s own social differences. TD students spoke about becoming less self-conscious and to focus one’s efforts on becoming the best version of oneself:

I think at university just from talking to people, a lot of them, like no one cares, I think everyone is just trying to get about their life as much as possible. I think it’s more to do with kind of being like you are your own person. . . (P28, TD)

Autistic students acknowledged the quantity and quality of social contact that one needed in order to maintain one’s wellbeing and not feeling pressured to be surrounded by people all the time. Individual differences in sociability were highlighted as some students actively tried to be involved but lacked success, and others acknowledged they preferred solitude but were aware how this can be unpopular among TD peers:

I don’t really like people that much, if I’m networking it’s a nightmare. Some people mistake that for unfriendliness, some people don’t like that sort of people and I understand, but in a way, that’s made making friends really hard. (P03, ASD)

Autistic students discussed the challenges they faced when trying to join a group at university. However, despite the difficulties, university seemed to bring on a sudden social awakening where they experienced for the first time the value of meeting and connecting with others, through pushing themselves to try out new things which they have found to be very enjoyable, and thus worth the amount of social effort they had put in. Autistic students spoke about being more active in their social relationships to initiate social engagements rather than passively taking part. However, autistic students also recognised how their social differences can be misunderstood by others and led to some degree of social exclusion and lack of close friendships, with one autistic student highlighting how she was ‘lucky’ because acceptance by others is ‘very unusual’ when you have autism.

#### Autonomy, competence and relatedness

We identified three themes from students’ responses that related to autonomy, competence and relatedness.

##### Routine and flexibility

Students expressed wanting to have more structured time and a routine at university to guide their activity planning:

I think if it was a 9-5 course, or normal teaching time, I don’t think I would have had the problems that I had for the whole 3 years, I think it would have been much easier. (P11, ASD)

Students talked about developing their own routine and ensuring that they integrated social and academic aspects of university life into their daily schedule. However, whereas TD students commented that it was important to enjoy the spontaneity of university life and have flexibility within one’s schedule to adapt to any changes, autistic students expressed a desire for clearer guidance and expectations for what to do during unstructured time on campus, which many struggled with.

##### Coping with uncertainty

Students expressed difficulties when coping with the uncertainties transitioning into and out of university. Having something familiar, such as living at home, having a good friend from school attending the same university or even having consistent hobbies can all be good ways to ground oneself despite all the changes:

I was quite lucky there, so I guess that sort of mediated that sort of negativity of not having my family and my close circles around. (P38, TD)

Students found the lack of clarity regarding academic expectations and how relationships can change or deteriorate over time to be particularly stressful. Attitudes towards stepping into the unknown differed among students, with some seeking the thrill of exploring unfamiliar territory, while others were reluctant to try out new things without guidance. Autistic students found the idea of being surrounded by strangers both in accommodation and at university to be particularly stressful, who commented that they could only truly unwind when they returned to parental home.

##### Family support

Compared to TD peers, many autistic students spoke about the importance of family providing a constant source of support throughout university. Parents served as advocates and liaisons for students:

It’s always easier to fight someone else’s battles than your own battles. . . my mum would usually end up fighting mine. (P01, ASD)

Autistic students found living at home to be a source of comfort, knowing that they had a safe space to return to at the end of the day provided reassurance despite facing challenges at university.

## Discussion

Following a critical realist approach, we identified aspects of both autistic and TD students’ university experience that are unique and shared across the conceptualisation of autonomy, competence and relatedness as defined by Ryan and Deci (2000) and provided evidence of self-determination when shaping the academic, daily living and socialisation aspects of university life. The flexible nature of a topic guide allowed us to follow-up and to clarify the role of *self*-versus-*other* in students’ ability to shape their own university experience. We highlight four main findings from this study in relation to wider literature, to help provide a better understanding of how self-determination is perceived by autistic students compared to their TD peers.

First, one striking comparison between autistic and TD students was the focus of self-determination efforts at university. Many autistic students commonly reported viewing academics to be the most important aspect of university life and had a strong sense of persistence and self-determination to succeed. Our study provides evidence that autistic students are aware of and able to flexibly use their cognitive strengths to their academic advantage at university, thus acting in an autonomous and competent manner (Field & Hoffman, 1999; Wehmeyer et al., 2010). Some autistic students were aware that their academic focus was at odds with the preference for socialisation shown by their TD peers, who were more engaged in a wider range of social settings and valued social life just as, if not more, important than academic studies.

However, while some autistic students viewed socialising to be a source of threat that could jeopardise their academic success if indulged in, others highlighted the importance of social connections at university beyond that of academics. Our seconding finding shows that some autistic students expressed similar perspectives with regards to social motivation and having the self-determination to initiate and establish relationships at university compared to their TD peers and found such autonomous behaviours resulted in improvements in social competence over time. For these autistic students, our finding resonates with a recent study which showed an association between self-determination and participation in structured social activities related to autistic adults’ interests (Kim, 2019), and such increased social opportunities helped them observe, evaluate and improve their social skills over time (Müller et al., 2008). Therefore, rather than lacking self-determination as suggested by previous studies (Wehmeyer et al., 2010; Wehmeyer & Shogren, 2008), we found many autistic students recognised that challenging themselves and developing social competencies was an important part of reaching their goal of relatedness and chose to compensate for their social communication differences by drawing upon their self-determination skills.

Autistic students who positively embraced new opportunities and developed meaningful social connections at university have been found to report better wellbeing, relative to those who had more difficulties in making friends (Bailey et al., 2020). Autistic students in this study expressed different degrees of sociability, drawing the distinction between feeling ‘alone’ and ‘lonely’, with the latter contributing to low mood and anxiety. Given that some autistic students found socialisation at university to be ‘necessary but exhausting’ (Van Hees et al., 2015), university stakeholders may consider supporting students to find the right balance of academic and social life for themselves at university. For example, universities may consider providing more psychoeducation on ways for maintaining one’s wellbeing at university and emphasise on the importance of setting wellbeing as a goal for successful university transition, beyond that of either social and/or academic success.

Third, many autistic students in this study expressed a preference for routine and stability and were more anxious when coping with changes in schedule and experienced greater emotion fluctuations. Compared to TD students, autistic students in the current sample reported a range of co-occurring mental health conditions, which present additional vulnerabilities. In the United Kingdom, mental health support from statutory healthcare services is not always readily accessible and often not well integrated with the more informal mental health support within the university such as counselling, wellbeing and peer support services (Batchelor et al., 2020; Byrom, 2018). Furthermore, although benefits from peer support has been noted among TD students (Byrom, 2018), challenges such as the need to overcome emotional barriers when disclosing one’s mental health difficulties to peers and fear of stigma, as well as concerns around the lack of professional training in peer support may reduce the adoption and efficacy of such alternative interventions (Batchelor et al., 2020). For autistic students, receiving mental health support that is adapted to cater for social communication differences in autism may be especially difficult (Camm-Crosbie et al., 2019), and there may be added issues around disclosure of autism diagnosis and both personal and external autism acceptance to consider in addition to mental health difficulties (Cage et al., 2018).

Autistic students’ experience of mental health may also be partially influenced by both the acceptance of their autism diagnosis by both themselves and others (Cage et al., 2018), such that greater personal and external autism acceptance was associated with lower depressive symptoms, though only the former was associated with reduced stress. Interestingly, anxiety was not associated with autism acceptance by self or others (Cage et al., 2018), which suggests other factors may be at play. In our study, autistic students may have a greater ‘tendency to react negatively on an emotional, cognitive and behavioural level to uncertain situations and events’ (Buhr & Dugas, 2009, p. 216), which is consistent with the reported increase of intolerance of uncertainty (IU) in autism (Boulter et al., 2014; Cai et al., 2018; Hwang et al., 2020; Wigham et al., 2015). IU encompasses a desire for the future to appear certain (desire for predictability) and difficulties making cognitive decisions or taking actions in the face of uncertainty (uncertainty paralysis) (Berenbaum et al., 2008; Birrell et al., 2011). IU might mediate the association of autism and anxiety (Boulter et al., 2014) and influence emotion regulation in autistic young people (Cai et al., 2018), where those with higher levels of IU were more likely to engage in maladaptive emotion regulation strategies such as suppression which inhibits appropriate emotional expression (Gross & Levenson, 1993), rather than adaptive strategies such as reappraising a situation to evaluate and modify its emotional impact (Lazarus & Alfert, 1964). Therefore, the need for autistic students to establish stable and consistent routines might be driven by a stronger sense of IU which increases their vulnerability to experience elevated levels of anxiety (Boulter et al., 2014). Given that we did not explicitly ask students about their IU, anxiety and emotion regulation strategies, future studies might investigate if and how any of such factors can interact with self-determination among both autistic and TD students at university.

Finally, self-determination and self-advocacy for autistic students to navigate and seek out appropriate mental health support and reasonable accommodations at university is somewhat inconsistent among our autistic students. The fourth main finding from our study is that autistic students highlighted that family continued to provide a constant source of support and comfort during university and continued to advocate for some students on their behalf. One autistic student in particular spoke about having the autonomy and competence to advocate for her friend at university, though struggled to do so for herself and turned to her mum for support. This suggests that rather than assuming that autistic students have poor advocacy skills in general, there may be additional barriers surrounding disclosure and *self*-advocacy at the university level. Some students hinted that coping with the emotional side of *self*-advocacy can be overwhelming and having a parent speaking on their behalf who understands their needs can be more efficient. One future direction may be for universities to consider developing a buddy scheme where two students may begin their university career by developing their advocacy skills through voicing each other’s needs, and receive skill-based training from university staff during their time at university to practice using their advocacy skills to voice their own needs. Helping students overcome the challenges of *self*-advocacy during university can further support their transition *out of* university such as seeking reasonable adjustments from employers during job interviews.

## Limitations

It should be noted that some of the challenges highlighted by autistic students in this study such as the reduced and less explicit structure at university, the contrast between better quality family support versus poor social integration, feelings of loneliness and lack of proactive campus-based support resonate with factors that were associated with the decision to drop-out of university by autistic students in recent quantitative and qualitative studies (Cage et al., 2020; Cage & Howes, 2020). Given that this study focused on self-determination at university as perceived by students, one limitation of this study is that we did not gather multi-informant reports from peers, university staff and family members to reflect on to what extent they supported students to develop and utilise their self-determination at university. Such perspectives are important to gather given that for almost half of the current sample of autistic students, family support helped students develop and assume their self-determination in their pursuit of university studies. Having a high and consistent level of family support when faced with varying quality of institutional and peer support may be especially relevant to maintain autistic students’ engagement at university (Cage & Howes, 2020) and considered as one part of the micro support system around the individual to secure better transition and retention outcomes (Cage et al., 2020).

This study utilised a TD student sample who did not have any current or past mental or physical health conditions. In contrast, many of our autistic students experienced co-occurring mental and physical health conditions that may have added unique challenges to their daily university life above and beyond that of their autism. Therefore, one limitation of this study is the lack of clarity around to what extent self-determination differences between autistic and TD students were related to having autism per se or additional challenges associated with other co-occurring conditions.

Another limitation is that the critical realist position adopted in this study might limit the extent to which our findings may be of use to research stemming from a more positivist background. However, anecdotal evidence from this study can help contextualise and extend beyond the quantitative differences in the capacity for self-determination from previous studies (Chou, Wehmeyer, Palmer, & Lee, 2017; Wehmeyer et al., 2010), and future studies may benefit from mixed-method designs (Chou, Wehmeyer, Shogren, et al., 2017; Wehmeyer & Kelchner, 1995; Wolman et al., 1994).

Finally, the use of the AQ as a means of screening and characterising autistic traits in both student samples also has caveats. Although the AQ has good sensitivity and specificity when used to discriminate between autistic individuals and TD controls (Booth et al., 2013), poorer specificity arises when the AQ is used to predict autism diagnosis in a clinically referred yet undiagnosed sample (Ashwood et al., 2016). When using the AQ to predict autism diagnosis in a sample referred to an autism diagnosis clinic in the United Kingdom, nearly two-thirds of students who scored below the clinical cut-off went on to receive an autism diagnosis, thus suggesting that the AQ did not predict autism diagnosis beyond chance level in a clinically referred sample (Ashwood et al., 2016). In the context of this study, the high sensitivity of the AQ may be helpful in detecting elevated autistic traits in a healthy TD sample, but its low specificity would mean that it cannot sufficiently prove that those who score below the cut-off in the clinical sample do not have an autism diagnosis. Therefore, we used elevated levels of autistic traits in the TD sample to exclude controls, though only used AQ scores to assess current levels of autism traits and not used to exclude autistic students if they scored below the cut-off. However, given that the AQ was the only means of characterising autistic traits/autism symptoms in this study, there may be some inaccuracies in self-reporting of symptoms. Future studies should seek to include and triangulate clinician and parent/carer assessments to further inform and verify autism diagnosis.

## Future directions

In addition to the future directions highlighted throughout the study, we outline two more future directions in response to the limitations identified in our study. Given that some of the students in this study were just beginning their undergraduate degrees, follow-up research can be an important next step to investigate whether and how students develop coping strategies as they progress through university or whether some of the current challenges discussed may predict their retention status in the near future. Future studies should also seek to compare and contrast how support from family, peers and educational staff may interact to support the development and maintenance of students’ self-determination in their ability to pursue, continue and complete higher education in the United Kingdom among autistic and TD students.

In order to understand the extent to which self-determination among autistic students at university was specific to challenges associated with autism, or related to their co-occurring conditions, future studies should seek to include a comparison group of non-autistic student with other conditions (mental and physical health, as well as specific learning difficulties). This will enable further exploration to understand the impact that having a diagnosis (other than autism) may have on students’ ability to act in a self-determined way at university and to better distinguish self-determination when specifically addressing autism related challenges at university among autistic students.

## Conclusion

In conclusion, the current findings help us understand self-determination that young people harness when navigating their development as young adults orienting to life goals. While as a highly individualistic process, shared experiences expressed by all students highlight that they provide a remarkable resource for themselves and others in similar positions. For autistic students, sharing their self-determination through the self-advocacy movement may support other incoming students to become more self-reliant and secure a successful transition *into, through* and *out* of university.

## Appendix 1

### Methods

#### Materials

##### Demographic questionnaire

Students reported their age, gender, ethnicity, autism and other past or current mental health, chronic physical illness or specific learning disability or developmental conditions. Students listed their pre-university performance by stating the number of A-levels (or equivalent) they completed and the grades received. Students reported their current degree of study and university attended, the current year of study they were in and living status.

##### AQ

The AQ is a 50-item self-report questionnaire which assesses five different domains of autistic traits, including social skills, attention switching, attention to detail, communication and imagination (Baron-Cohen et al., 2001). Students rated the extent to which they agree with each statement, which is subsequently scored either 0 or 1 depending on whether or not the autistic trait has been endorsed. The AQ has good internal consistency and test re-test reliability (Baron-Cohen et al., 2001) and has a screening cut-off score of ⩾26 and clinical cut-off score of ⩾32.

### Results

#### Interview format and duration

All students completed the interview either in person or via the phone (Table 1), which lasted between 20 to 50 min for TD students and 20 min to 1 h for autistic students. Comparing the word count from interview transcripts, there were no statistically significant differences (*t*(34) = 0.60, *p* = 0.55) between interview length for autistic students (*M* = 5878.33, *SD* = 1595.90) and that of TD students (*M* = 5575.72, *SD* = 1409.94). Given that one autistic student helped to improve the topic guide clarity during pilot phase, both student groups perceived the interview schedule to be clear and easy to follow, and we did not make any further adaptations.

## Appendix 2

Questions from the interview topic guide:

### Warm up questions

1. Which course are you studying?
2. Which year of study are you in? How long is your programme?
3. How did you reach the decision to start university? How old were you?

### Interview questions

1. Thinking back to first starting university, what was transitioning to university like for you?
  PROMPT:
  *Think back to moving in, Freshers’ Week, introductory lectures, meeting your flat mates for the first time?*
2. Were there any previous experiences, or things that you have done, which you felt were helpful in preparing you for making the transition to university?
3. How has university life been like for you?
  PROMPTS:
  *Can you tell me about some of the positive and negative experiences you have had?*
  *Has anything changed since first transitioning to university? How have things changed?*
  *Prompt for academic, daily living, and social domains.*
4. To what extent do you feel like your university life is being shaped by you? (or in other words, to what extent do you feel like you are in control of your university life?)
  PROMPTS:
  *Which personal qualities or strengths do you think have helped you?*
  *Are there other people that have helped you along the way?*
  *Any formal/informal support? If yes, What has helped you in seeking support?*
5. How do you think your university life might compare to other students?
  *Prompt for academic, daily living, and social domains.*
6. Are there things you wish would be different in your university life? What are they?
  *Prompt for academic, daily living, and social domains.*
7. Autistic students only: In what ways do you think autism has had an impact on your university life?
8. What do you think life might be like when you graduate from your current degree or when you leave university?
  PROMPT:
  *How do you think it will compare to your experience of transitioning to university?*
9. Do you think there are things you have done, or skills you have gained during your time at university that will help prepare you for transitioning out of university?
10. Is there anything else about your experience of university life (transition to and from university) that you would like to add/that you think is important and we have not mentioned yet?

## Notes

1. A recent publication (Kenny et al., 2016) that investigated language preference when referring to autism found that autistic individuals and their families and friends prefer to use identity-first language when referring to autism (i.e. autistic individual), rather than person-first language (i.e. person with autism), though the latter was more commonly used by professionals. In this article, we will use identity-first language.
